# Investigation of the prognostic predictive value of serum lipid profiles in amyotrophic lateral sclerosis: roles of sex and hypermetabolism

**DOI:** 10.1038/s41598-022-05714-w

**Published:** 2022-02-03

**Authors:** Ryutaro Nakamura, Mika Kurihara, Nobuhiro Ogawa, Akihiro Kitamura, Isamu Yamakawa, Shigeki Bamba, Mitsuru Sanada, Masaya Sasaki, Makoto Urushitani

**Affiliations:** 1 Department of Neurology, Shiga University of Medical Science, Tsukinowa, Seta, Shiga Japan; 2 Division of Clinical Nutrition, Shiga University of Medical Science, Tsukinowa, Seta, Shiga Japan

**Keywords:** Neuroscience, Diseases, Medical research, Neurology, Risk factors

## Abstract

The prognostic predictive value of lipid profiling in amyotrophic lateral sclerosis (ALS) remains unclear. Here, we aimed to clarify the value of the levels of serum lipids, including high-density lipoprotein cholesterol (HDL), low-density lipoprotein cholesterol (LDL), and triglycerides (TG), for predicting the prognosis in ALS. This was a single-center retrospective study of 78 patients with ALS. The serum lipid profiles at the first hospital visit after symptom onset were analyzed to determine the correlations of lipids with survival and physical parameters, including nutritional, respiratory, and metabolic conditions. The cutoff level for high HDL was defined as the third quartile, while that of low LDL and TG, as the first quartile. Hypermetabolism was defined as the ratio of resting energy expenditure to lean soft tissue mass ≥ 38 kcal/kg. High HDL was an independent factor for poor prognosis in all patients (hazards ratio [HR]: 9.87, *p* < 0.001) in the Cox proportional hazard model, including %vital capacity and the monthly decline rate in body mass index and the Revised Amyotrophic Lateral Functional Rating Scale score from symptom onset to diagnosis. Low LDL was a factor for poor prognosis (HR: 6.59, *p* = 0.017) only in women. Moreover, subgroup analyses with log-rank tests revealed that the prognostic predictive value of high HDL was evident only in the presence of hypermetabolism (*p* = 0.005). High HDL predicts poor prognosis in all patients, whereas low LDL, only in women. Hypermetabolism and high HDL synergistically augment the negative effect on prognosis.

## Introduction

Amyotrophic lateral sclerosis (ALS) is a neurodegenerative disease of the upper and lower motor neurons, which leads to lethal respiratory failure within a few years^1^. Although curative treatments are awaited, a high-calorie diet slows disease progression in patients exhibiting rapid progression^2^, and noninvasive positive pressure ventilation decelerates the respiratory functional decline^3^. Since early intervention with these therapies would be beneficial, the identification of accessible biomarkers with high diagnostic and prognostic value is required.

Previous studies have shown that hypermetabolism is a feature of ALS and is a poor prognostic factor^4–6^. On the other hand, Steyn et al. demonstrated that increased fatty acid oxidation enhances resting energy expenditure, which leads to slower progression in superoxide dismutase (SOD) mice^7^. We previously showed that patients with hypermetabolism, defined as the ratio of measured resting energy expenditure (mREE) to lean soft tissue mass (LSTM) (mREE/LSTM) ≥ 38, exhibited significantly shorter survival than patients with normal metabolism only when their body mass index (BMI) was ≥ 19.8^8^. Contrarily, patients with hypermetabolism and BMI < 19.8 exhibited a clear trend of prolonged survival. This discrepancy led us to formulate the BMI-muscle metabolism index (BMM index), defined as (BMI − 19.8) × (mREE/LSTM − 38); a high BMM index is an independent factor for poor prognosis. Furthermore, we showed that low body fat percentage was associated with poor prognosis^8^, as reported elsewhere^9^. These findings may indicate that the prognostic value of hypermetabolism depends on nutritional status and that lipid metabolism plays a crucial role in determining the prognosis of ALS.

Cholesterol is attracting significant attention as a predictive factor for prognosis and a pathomechanistic mediator in ALS. There are reports indicating that high triglycerides (TG)^10–12^ and high low-density lipoprotein cholesterol (LDL)^13–15^ are favorable prognostic markers based on univariate analyses, while others argue against this relationship^11,12^. The relevance of high-density lipoprotein cholesterol (HDL) is also controversial. A considerable number of studies^11,12,17–19^ have reported that HDL was not associated with survival. Ingre et al. examined lipid profiles at diagnosis, and they revealed that high LDL was an independent factor for favorable prognosis based on a multivariable analysis^20^. Patients with high HDL had shorter survival than patients with low HDL per the results of log-rank tests, although the difference was not significant (*p* = 0.06). Barrons et al. reported that both LDL and HDL were positively associated with the Revised Amyotrophic Lateral Sclerosis Functional Rating Scale (ALSFRS-R) score^18^. Therefore, the prognostic value of lipid profiles might be affected by disease progression, which may explain the discrepancies among previous studies.

In this study, we aimed to investigate the results of serum lipid profiling performed at an earlier stage of ALS, including at the pre-diagnostic stage in referring hospitals, in order to exclude confounding effects with the goal of identifying prognostic factors for preventing disease progression. Through an analysis of sex-based differences, we found that early lipid profiles predict the prognosis of ALS. Moreover, hypermetabolism was found to govern the predictive value of lipids for ALS prognosis, which could reconcile the previous controversies.

## Materials and methods

### Patients and ethical considerations

This retrospective study was conducted in a single-center and was approved by the Ethics Committee of Shiga University of Medical Science Hospital (approval no.: R2020-171). We included patients who visited the Shiga University of Medical Science Hospital between March 2016 and September 2021. All patients fulfilled the revised El Escorial criteria for probable or definite ALS^21^.

### Clinical parameters

The monthly decline rate in BMI from the onset to the first visit (ΔBMI) was calculated as (premorbid BMI − BMI at the first visit)/(duration in months). Disease progression was estimated by the monthly decline rate in the ALSFRS-R score from symptom onset to diagnosis (ΔALSFRS-R), as (48 − ALSFRS-R score at admission)/(time from onset to diagnosis in months)^22^. We also assessed age, the region of the initial symptom, sex, statin use, and %vital capacity (%VC; %).

### Blood test

We obtained data regarding serum HDL (mg/dL), LDL (mg/dL), and TG (mg/dL) levels from the results of the first blood test performed in our institute or in a previous hospital after the patients experienced the first symptom. The cutoff value of HDL was defined as the third quartile and that of LDL and TG, as the first quartile. To monitor the chronological change in lipid levels, we performed blood sampling at 3 to 6 months after the first test. We did not exclude data obtained in the non-fasting condition or data calculated using the Friedewald equation since fasting is not routinely required for lipid profiling, and calculated LDL is comparable to its direct measurement during non-fasting lipid profiling^23^.

### Energy metabolism and body composition

We obtained data regarding mREE (kcal) through indirect calorimetry (Aeromonitor® AE310S, Minato Medical Science Co., Osaka, Japan.), and LSTM (kg) was measured through a bioelectrical impedance analysis with a body composition analyzer (InBody S10; InBody, Tokyo, Japan). We used indirect calorimetry as reported previously^8,24^. In this study, as an indicator of the metabolic status, we used mREE/LSTM, which approximately reflects the skeletal metabolic status during the resting state; additionally, the relationship of this indicator with hypermetabolism was described in previous studies^4,25^. We defined hypermetabolism as mREE/LSTM ≥ 38, according to our previous report^8^. The ratio of mREE to calculated REE (cREE) obtained using the Harris-Benedict equation^26^ is universally used to estimate metabolism^4–6,26^. However, cREE might be overestimated in Japanese people^27^, and thus, we used our own formula in order to exclude the effects caused by population-based differences. The BMM index was used as a prognosis prediction formula, defined as (BMI − 19.8) × (mREE/LSTM − 38), as we have reported previously^8^.

### Statistical analysis

We summarized variables as the median and interquartile range and used Fisher's exact test or the Mann–Whitney U test for data comparison between the different groups. The relationship among the lipids in each sex was evaluated using the Spearman rank-correlation method. The interval change in lipid data calculated during the follow-up blood test was analyzed using the Wilcoxon signed-rank test. We defined the endpoint as the time of death or tracheotomy and the survival time as the duration from the first symptom to the aforementioned endpoints or censoring time. The censoring time for the follow-up period was at the end of September 2021. Survival was evaluated using Kaplan–Meier curves and log-rank tests. The absolute risks of high HDL, low LDL, and low TG were estimated by Cox proportional hazards (PH) regression analyses of each lipid profile, ΔALSFRS-R, ΔBMI, and %VC in each sex. The risk associated with each of the lipids in all the patients was investigated by Cox PH regression analyses of each lipid in relation to the confounding factors, including age, ΔALSFRS-R, ΔBMI, the onset region (bulbar type), sex, and %VC. We also performed Cox analyses in the models including these factors and the BMM index. Statistical analyses were two-sided, and statistical significance was set at *p* ≤ 0.05. All analyses were performed using EZR version 1.53 (Saitama Medical Center, Jichi Medical University, Tochigi, Japan), which is based on R and R commander (The R Foundation for Statistical Computing, Vienna, Austria)^28^.

### Patient consent for publication

We applied the opt-out method and obtained informed consent for this study, which was performed in accordance with the ethical standards laid down in the 1964 Declaration of Helsinki and its later amendments. The participants consented to the submission of this study to the journal.

### Ethical approval

This study was approved by the Ethics Committee of Shiga University of Medical Science Hospital (approval no.: R2020-171).

### Provenance and peer review

Not commissioned; externally peer reviewed.

## Results

### High HDL in both sexes and low LDL in women are poor prognostic factors in ALS

During the study period, 79 patients were diagnosed with ALS, and we included 78 patients who had lipid profile data. Their demographic and clinical data are presented in Table 1. Eleven of the 38 male patients and 15 of the 40 female patients had bulbar-type ALS. Only one patient underwent a genetic test and had the L106V mutation in the superoxide dismutase-1 gene.

**Table 1:** Demographic and clinical data.

		All	Male patients	Female patients	
	n	78	38	40	
Age (y)	78	71 [66, 75]	70 [64, 75]	73 [67, 75]	
ALSFRS-R score	69	41.00 [36, 44]	38 [35, 43]	42 [39, 44]	
ΔALSFRS-R	69	0.56 [0.27, 1.00]	0.56 [0.26, 1.00]	0.52 [0.31, 0.87]	
BMI at the first visit (kg/m2)	78	21.7 [18.7, 24.1]	22.1 [20.0, 24.5]	20.8 [18.5, 23.4]	
ΔBMI	74	0.19 [0.01, 0.33]	0.18 [0.01, 0.43]	0.20 [0.07, 0.31]	
Bulbar type (yes)		26/78 (33%)	11/38 (29%)	15/40 (38%)	
Hypermetabolism (yes)		25/53 (47%)	9/24 (38%)	16/29 (55%)	
mREE/LSTM (kcal/kg)	53	37.1 [34.5, 41.2]	35.6 [34.0, 39.2]	38.7 [34.5, 42.6]	
Statin use (yes)		21/78	7/38 (18%)	14/40 (35%)	
%VC (%)	70	88 [78, 95]	90 [81, 99]	87 [72, 95]	
Endpoint		41/78	22/38	19/40	
Blood test for the first time					p*
HDL (mg/dL)	78	63 [50, 75]	60 [49, 71]	65 [57, 76]	0.175
High HDL		23	10/38 (26%)	13/40 (33%)	0.62
LDL (mg/dL)	78	115 [96, 139]	111 [80, 125]	129 [104, 143]	**0.008**
Low LDL		19	10/38 (26%)	9/40 (23%)	0.79
TG (mg/dL)	77	109 [74, 143]	120 [76, 164]	103 [73, 136]	0.51
Low TG		20	9/38 (24%)	11/39 (28%)	0.80
Interval between the onset and					
blood test (months)	78	10 ^6,15^	11 ^6,18^	9.00 [6.00, 13]	

Data are presented as median [interquartile range]. Due to missing values, the number corresponding to each factor does not necessarily add up to the total number of 78. *P-value is based on Fisher's exact test or the Mann–Whitney U test.

ALSFRS-R, Revised Amyotrophic Lateral Functional Rating Scale; BMI, body mass index; BMM index, BMI muscle metabolism index; mREE, measured resting energy expenditure; LSTM, lean soft tissue mass; VC, vital capacity; HDL, high-density lipoprotein; LDL, low-density lipoprotein; TG, triglycerides.

Significant values are in [bold].

LDL was significantly higher in women than in men (*p* = 0.008), and there were no patients with TG > 400. We determined the following cutoff values: HDL, 70 mg/dL in men and 75 mg/dL in women; LDL, 80 mg/dL in men and 100 mg/dL in women; and TG, 75 mg/dL in both men and women. HDL was negatively associated with TG in men (rs = -0.57, *p* < 0.001) and women (rs = − 0.39, *p* = 0,015), and LDL was positively associated with TG in men (rs = 0.40, *p* = 0.012) (Supplemental Fig. 1). The follow-up blood test in 40 patients (18 men and 22 women) revealed that HDL significantly decreased in both men (*p* = 0.010) and women (*p* = 0.023) and that LDL significantly decreased only in women (*p* = 0.030) (Supplemental Fig. 2). No patients began treatment with statins from the first test to the follow-up one. The other patients did not have lipid profile data at 3 to 6 months from the first test.

**Figure 1:**
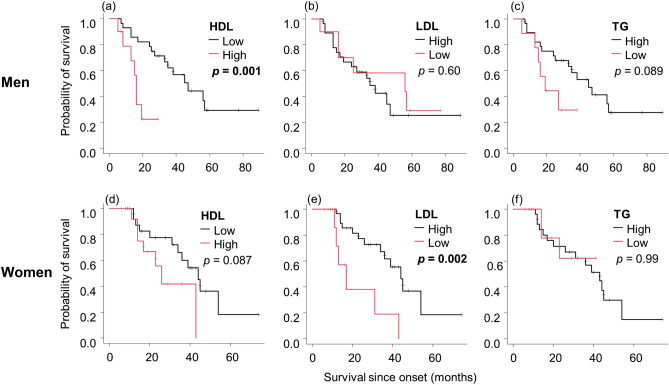
The relationship between the lipid profiles and survival in each sex. Kaplan–Meier analyses and log-rank tests revealed that men with high HDL (**a**) and women with low LDL (**e**) had significantly shorter survival. Men with low TG (**c**) and women with high HDL (**d**) showed the same trend, although not significant. There was no significant relationship in the other groups (**b**, **f**). Abbreviations: HDL, high-density lipoprotein; LDL, low-density lipoprotein; TG, triglycerides.

**Figure 2:**
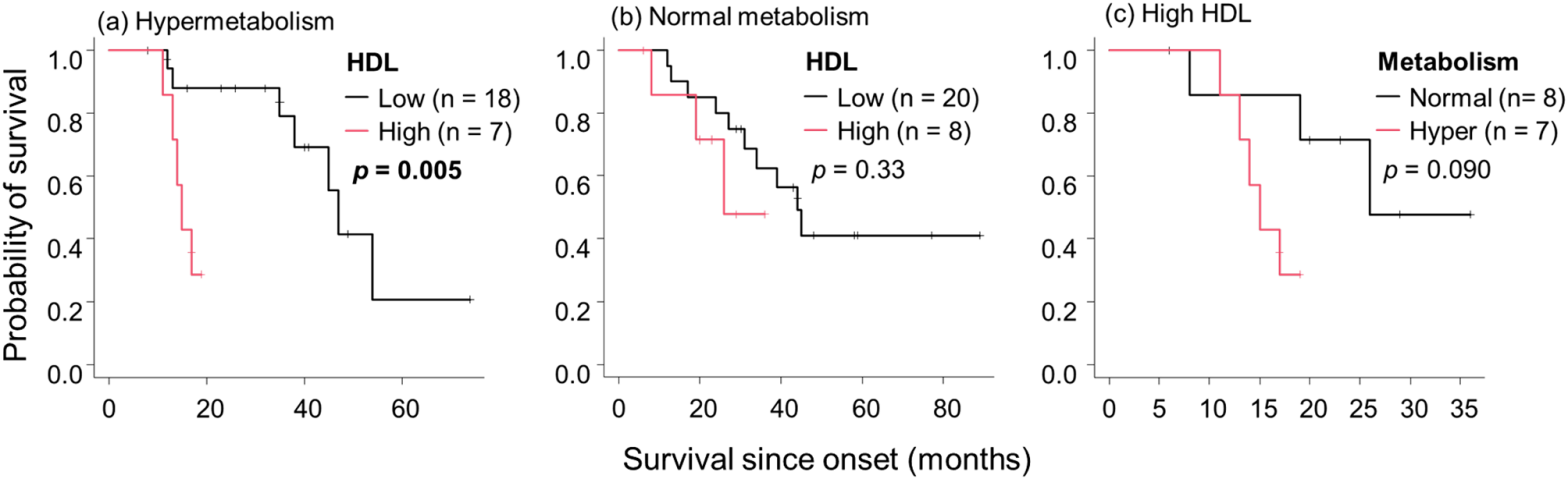
The relationship between HDL and hypermetabolism in terms of survival. Kaplan–Meier analyses and log-rank tests revealed that among patients with hypermetabolism, those with high HDL had significantly shorter survival (**a**), which was not the case in patients with normal metabolism (**b**). Patients with hypermetabolism had shorter survival than patients with normal metabolism in the high HDL group, although this finding was not significant (**c**). Abbreviations: HDL, high-density lipoprotein.

Subsequently, we analyzed the relationship between lipid components and various clinical factors in each sex. ΔBMI was significantly higher in men with low TG than in those with high TG (*p* = 0.020, Supplemental Fig. 3). Men with high HDL and women with low LDL exhibited a non-significant but similar trend (*p* = 0.084 and *p* = 0.073, respectively). The ΔALSFRS-R was significantly higher in men with high HDL than in those with low HDL (p = 0.006, Supplemental Fig. 4). Men with low TG showed a similar but non-significant trend (*p* = 0.087). %VC was significantly lower in men with low TG than in those with high TG (*p* = 0.032, Supplemental Fig. 5). A similar but non-significant trend was observed in men with high HDL and women with low LDL (*p* = 0.058 and *p* = 0.086, respectively). Lipid profiles were not significantly correlated with mREE/LSTM in either male or female patients (Supplemental Fig. 6). However, male patients with high HDL and low TG had a significantly higher BMM index, which is our previously reported prognosis-predictive formula^8^ (Supplemental Fig. 7).

**Figure 3:**
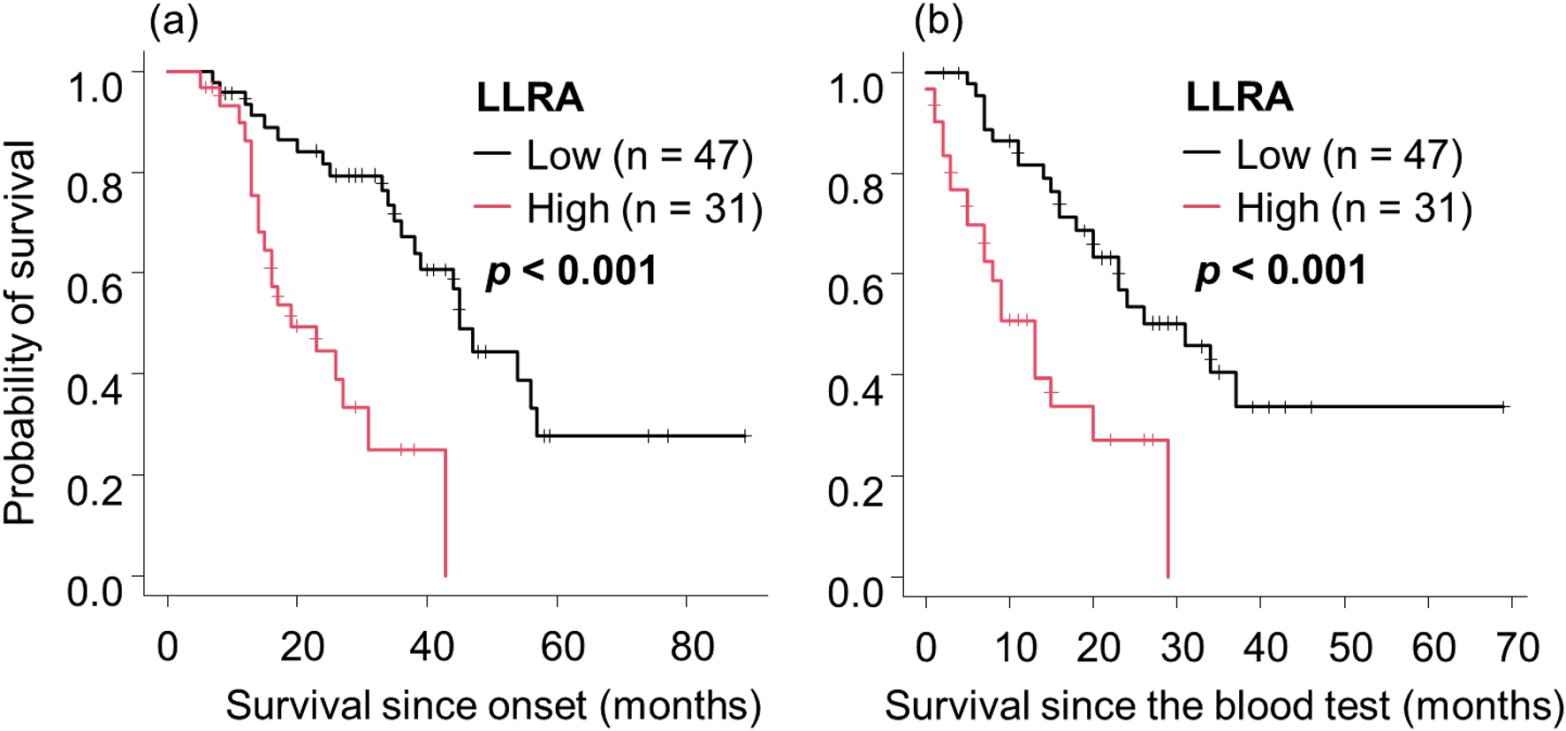
The survival of patients stratified by LLRA. Kaplan–Meier analyses and log-rank tests revealed that patients with high LLRA had significantly shorter survival since onset and since the blood test than patients with low LLRA (**a**,**b**). Abbreviations: ALS, amyotrophic lateral sclerosis; LLRA, lipid-linked risk for ALS.

**Figure 4:**
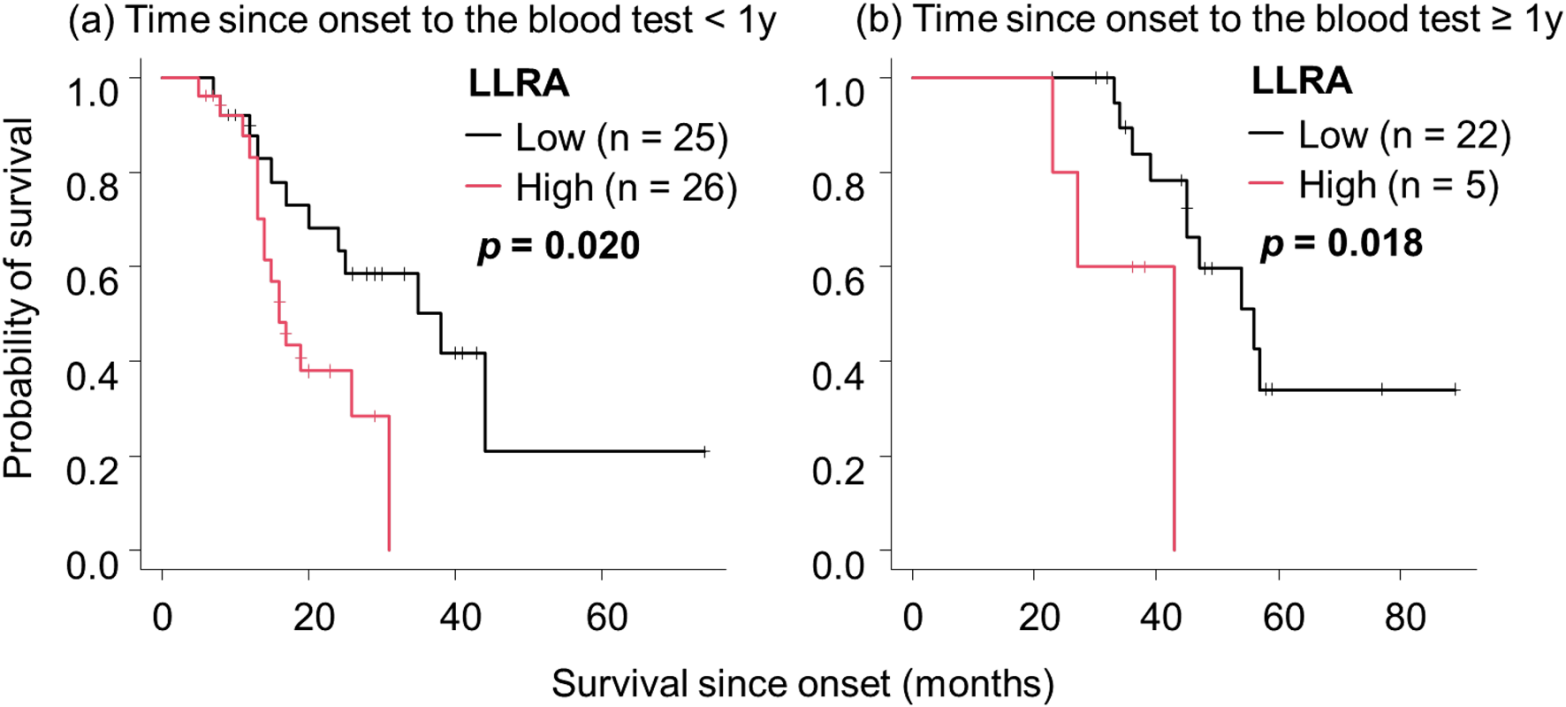
Significance of LLRA as a predictive factor for poor prognosis. Kaplan–Meier analyses and log-rank tests revealed that LLRA was a significant factor for poor prognosis in patients whose time since onset to the blood test was shorter than 1 year (**a**) and in those with an interval longer than 1 year (**b**). Abbreviations: ALS, amyotrophic lateral sclerosis; LLRA, lipid-linked risk for ALS.

**Figure 5:**
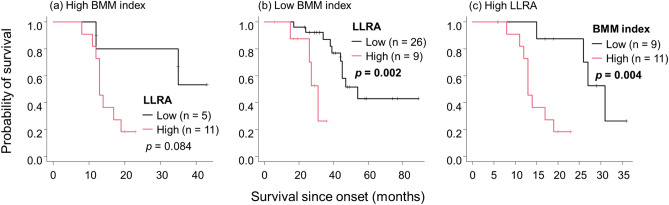
The relationship between the BMM index and LLRA in terms of survival. In the high BMM index group, patients with high LLRA had shorter survival than those with low LLRA although the difference was not significant (**a**), whereas the difference was significant in the low BMM index group (**b**). In the high LLRA group, patients with a high BMM index had significantly shorter survival than those with a low BMM index (**c**). Abbreviations: BMI, body mass index; BMM, BMI-muscle metabolism index; ALS, amyotrophic lateral sclerosis; LLRA, lipid-linked risk for ALS.

Next, we investigated the relationship between survival and lipid profiles. Men with high HDL had significantly shorter survival than those with low HDL (*p* = 0.001, Fig. 1). Women showed a similar but non-significant trend (*p* = 0.087). On the other hand, women with low LDL exhibited significantly shorter survival than those with high LDL (*p* = 0.002), which was not the case in men. Men with low TG showed a trend predicting poor prognosis compared with those with high TG; however, the difference was not significant (*p* = 0.089). There was no association between the survival of women and TG levels. We also examined the association between survival and lipid levels using the cutoff values described in the study by Ingre et al.^20^. The approximate cutoff values based on log-rank tests were as follows: HDL, 55 mg/dL; LDL, 100 mg/dL; and TG, 70 mg/dL. Men with high HDL had significantly shorter survival than those with low HDL (*p* = 0.034) when considering a lower cutoff HDL level of 55 mg/dL, and women showed a similar but non-significant trend (*p* = 0.114) (Supplemental Fig. 8). Men with LDL < 100 mg/dL did not show shorter survival than those with LDL ≥ 100 mg/dL. Therefore, the cutoff LDL levels in women and cutoff TG levels in both sexes in our study were comparable to those in the study by Ingre et al.

We further analyzed the role of metabolism and lipid profiles in the prognosis of ALS. Among patients with hypermetabolism, those with high HDL showed a significantly shorter survival than those with low HDL (*p* = 0.005, Fig. 2); this was not seen in normometabolic patients. Hypermetabolism was an apparent but non-significant risk factor for shorter survival in patients with high HDL (*p* = 0.090). Similarly, in the hypermetabolism group, patients with low LDL showed a trend of shorter survival compared with those with high LDL (*p* = 0.090, Supplemental Fig. 9). Hypermetabolism was not correlated with low TG in terms of survival (Supplemental Fig. 10).

Based on the Cox PH models for each sex, which included each lipid profile (high HDL, low LDL, or low TG), ΔALSFRS-R, ΔBMI, and %VC, we found that high HDL was an independent factor for poor prognosis in both men and women, while low LDL was a factor only in women (Table 2). Moreover, Cox PH models for the entire patient group, which included lipid profile data, age, ΔALSFRS-R, ΔBMI, bulbar type, sex, and %VC, revealed that HDL was an independent factor for poor prognosis. Notably, the significance was not affected by the addition of the BMM index to the model.

**Table 2:** Cox PH models.

		Male patients		Female patients		All			
		Adjusted HR	p*	Adjusted HR	p*	Adjusted HR†	*p*†	Adding BMM index‡	
								Adjusted HR‡	*p*‡
HDL	High vs. low	**8.43 (1.67–42.5)**	**0.010**	**16.2 (2.15–121.4)**	**0.007**	**9.87 (3.10–31.5)**	** < 0.001**	**10.2 (1.83–56.8)**	**0.008**
LDL	Low vs. high	0.55 (0.15–2.09)	0.38	**6.59 (1.41–30.7)**	**0.017**	1.00 (0.99–1.01)	0.77	0.65 (0.16–2.61)	0.54
TG	Low vs. high	0.35 (0.10–1.24)	0.104	NA	NA	0.66 (0.20–2.14)	0.49	0.86 (0.21–3.43)	0.83

P-values are based on Cox PH models. We could not include TG in the Cox analysis of women due to the small number of events.

*These models included each lipid profile (high HDL, low LDL, or low TG) , ΔALSFRS-R, ΔBMI, and %VC.

^†^These models included age, ΔALSFRS-R, ΔBMI, bulbar type, sex, and %VC.

^‡^These models included lipid profile data, BMM index, age, ΔALSFRS-R, ΔBMI, bulbar type, sex, and %VC. PH, proportional hazards; HR, hazard ratio (95% confidence interval); NA, not available; ALSFRS-R, Revised Amyotrophic Lateral Functional Rating Scale; BMI, body mass index; BMM index, BMI muscle metabolism index; VC, vital capacity; HDL, high-density lipoprotein; LDL, low-density lipoprotein; TG, triglycerides.

Significant values are in [bold].

### Lipid-linked risk for ALS (LLRA) is a strong predictive factor for ALS

Based on our results, we determined that the presence of either high HDL in all patients or low LDL in female patients was a lipid-linked risk factor for ALS (LLRA). We then stratified patients into high or low LLRA groups and evaluated the significance of LLRA in relation to various clinical parameters (Table 3).

**Table 3:** Demographic and clinical data in relation to LLRA.

	LLRA		
	Low	High	p*
n	47	31	
Age (y)	69 [66, 75]	74 [63, 76]	0.27
ALSFRS-R score	41 [37, 44]	40 [36, 43]	0.6
ΔALSFRS-R	0.48 [0.20, 0.87]	0.71 [0.38, 1.14]	0.058
BMI at the first visit (kg/m2)	21.6 [19.9, 24.7]	21.7 [17.6, 23.1]	0.33
ΔBMI from onset to the first visit/month	0.13 [0.00, 0.28]	0.27 [0.18, 0.48]	**0.008**
BMM index	-3.7 [-14.6, 0.0]	1.5 [-1.9, 4.8]	**0.006**
Bulbar type (yes)	12/47 (25%)	14/31 (45%)	0.089
Fasting (yes)	9/47 (19%)	2/31 (6%)	0.184
Hypermetabolism (yes) †	15/31 (48%)	10/22 (45%)	1
Sex (men)	25/47 (53%)	13/31 (42%)	0.36
Time since onset to the blood test (months)	11.0 [7.0, 16.0]	9.0 [5.0, 11.0]	0.056
Time since onset to the blood test ≥ 1 year	22/47 (47%)	5/31 (16%)	**0.007**
%VC	90.6 [84.7, 98.4]	82.7 [59.2, 92.4]	**0.012**

Data represent the median value [interquartile range].

*p < 0.05, by Fisher's exact test or the Mann–Whitney U test.

^†^Hypermetabolism was defined as mREE/LSTM ≥ 38 kcal/kg. LLRA, lipid-linked risk for ALS; ALSFRS-R, Revised Amyotrophic Lateral Functional Rating Scale; BMI, body mass index; BMM index, BMI-muscle metabolism index; VC, vital capacity.

Significant values are in [bold].

Patients with high LLRA had a significantly higher ΔBMI, higher BMM index, and lower %VC than those with low LLRA. Patients with high LLRA exhibited significantly shorter survival from the time of both the clinical onset (*p* < 0.001, Fig. 3a) and blood test (*p* < 0.001, Fig. 3b), indicating the predictive role of high LLRA for poor prognosis in ALS. Regardless of whether there was a delay of > 1 year between the onset and blood test, the survival of patients with high LLRA was significantly shorter than that of those with low LLRA (Fig. 4). The percentage of patients who took the blood test > 1 year after the onset was smaller in the high LLRA group than in the low LLRA group (Table 3). Patients with high LLRA had significantly shorter survival than those with low LLRA regardless of fasting before blood sampling or the use of the Friedewald equation (Supplemental Fig. 11).

We then investigated the relationship between LLRA and the BMM index in terms of survival. In the low BMM index group, patients with high LLRA showed significantly shorter survival than those with low LLRA (Fig. 5b). Patients with a high BMM index also showed an apparent but non-significant reduction in survival (Fig. 5a). Moreover, in the high LLRA group, the survival of patients with a high BMM index was significantly shorter than that of patients with a low BMM index.

### Statin use did not affect the survival of ALS

In our study, twenty-one patients used statins for dyslipidemia, and their lipid profiles were summarized in the Supplemental Table. The percentages of high HDL and high LLRA were significantly lower in patients using statins than in those without (*p* = 0.024). Other lipid profiles were not different between the two groups. Statin use did not affect the survival (Supplemental Fig. 12a) in all patients, but the low LDL in women was also a poor prognostic factor in patients using statins (Supplemental Fig. 12c). Only two of seven men who use statin had low LDL, which was insufficient to validate the prognostic value.

## Discussion

We have shown that high HDL was an independent factor for poor prognosis in patients with ALS (Table 2). Moreover, there was a sex-based difference in the predictive value of LDL. Furthermore, the combination of the BMM index and presence of either high HDL in both sexes or low LDL in female patients (namely LLRA) improved the prognostic predictive value (Fig. 5).

Several studies have argued against the correlation between HDL and survival in ALS^10–12,16,17,29^. One possible explanation for the discrepancy between these reports and ours is the timing of blood sampling after the clinical onset. Ingre et al. analyzed the blood data obtained at the time of diagnosis and revealed that low LDL was an independent factor for poor prognosis based on a multivariate analysis^20^. In this study, we collected the first set of blood data after the appearance of the initial symptom; therefore, we could analyze the serum as early as 10 months after the clinical onset. Another factor that enabled us to confirm the predictive value of HDL was our cutoff value for high HDL. The cutoff values defined as the third quartile in our cohort were 70 mg/dL in men and 75 mg/dL in women (Table 1), which were higher than those in previous studies ^17,20^. Indeed, the lower cutoff value of 55 mg/dL used in the study by Ingre et al. weakened the trend of patients with high HDL exhibiting shorter survival than those with low HDL (Supplemental Fig. 8). Notably, HDL decreased along with disease progression in ALS (Supplemental Fig. 2) and was significantly correlated with the ALSFRS-R score ^18^. Thus, an earlier analysis of the lipid profile is necessary to predict the prognosis of ALS.

We also found that sex-based differences may be responsible for the discrepancy in results among studies. Low LDL was not a poor prognostic factor in men (Fig. 1) in contrast to in women. Furthermore, the relationship between lipid profiles and clinical features, including ΔALSFRS-R, ΔBMI, and %VC, differed depending on sex. The mechanism underlying sex-based differences in the roles of lipids in ALS prognosis remains elusive. Previous animal studies have reported sex-based differences in the phenotypes of ALS models. For instance, the inactivation of liver X receptor β caused lipid accumulation and loss of motor neurons in the spinal cord only in male mice^30^. Hormonal factors could also be involved, as reported by Choi et al. who demonstrated the protective role of estrogen in ALS^31^.

The relationship between lipid metabolism and survival in ALS remains unclear. Of course, malnutrition and respiratory functional decline can be the cause, since male patients with high HDL and female patients with low LDL showed a rapid decline in BMI and lower %VC (Supplemental Figs. 3 and 5). Nevertheless, multivariate analyses that included ΔBMI, %VC, and ΔALSFRS-R validated the finding that both high HDL in all patients and low LDL in female patients were independent factors for poor prognosis (Table 2). Furthermore, ΔALSFRS-R, ΔBMI, and %VC were not significantly different regardless of high or low HDL in women (Supplemental Figs. 3–5), which may indicate that high HDL itself affects the prognosis. It has been reported that HDL activates the mammalian target of rapamycin-1 (mTORC1) via phosphatidylinositol 3-kinase (PI3K)-Akt signaling in muscles^32,33^ and adipocytes^34^, resulting in autophagy inhibition ^34^. This pathway may ultimately accelerate the progression of ALS through the accumulation of ALS-linked pathogenic proteins, such as trans-activation response (TAR) deoxyribonucleic acid (DNA)-binding protein of 43 kDa (TDP-43). In addition, HDL increases glycolysis and glucose uptake into the muscle via the activation of PI3K-Akt signaling in skeletal muscle^32,33^. The upregulation of glycolysis plays a protective role in terms of energy supply in motor neurons but not in muscles^35^. The induction of muscle glycolysis by HDL might be detrimental for the ALS prognosis since the energy supply reportedly shifts from glycolytic to lipid metabolism, leading to beta-oxidation in muscles during starvation in ALS^36^.

Hypermetabolism has recently been implicated in the poor prognosis of ALS^4–6^. In this study, patients with hypermetabolism along with high HDL exhibited significantly shorter survival than those with low HDL. By contrast, high HDL in normometabolic patients did not alter prognosis (Fig. 2a, b). Furthermore, in the high HDL group, patients with hypermetabolism showed a clear but non-significant trend of shorter survival than normometabolic patients (p = 0.09) (Fig. 2c). These results indicate that hypermetabolism and high HDL synergistically augmented the negative effect on ALS prognosis; specifically, increased glycolysis in muscle might underlie poor prognosis given that HDL might stimulate the glycolytic system in skeletal muscle. Furthermore, in the hypermetabolism group, patients with high LDL showed a trend of prolonged survival compared to those with low LDL (Supplemental Fig. 9). It is possible that lipid metabolism may play a protective role in hypermetabolic conditions in ALS.

We also examined the prognostic value of lipid profiles in 21 patients who were using statins. As expected, the proportion of patients with high HDL was smaller among those using statins than among those not using these medications (Supplemental table); this might be because patients with dyslipidemia tend to have low HDL^37^. The number of patients with high HDL among those using statins was not sufficient to validate the prognostic value. However, female patients with low LDL exhibited significantly shorter survival than those with high LDL in the statin group, despite the small number of patients (Supplemental Fig. 12). Our results highlight the potential risk of lipid-lowering therapy in female patients. However, future studies with larger sample sizes are required to confirm this.

This study has several limitations. First, this was a single-center retrospective study in Japan, and the number of patients was relatively small. Second, most blood tests in this cohort were performed without fasting. Indeed, many previous studies have included only patients who fasted overnight. However, per the European Atherosclerosis Society and European Federation of Clinical Chemistry and Laboratory Medicine, a lipid assessment does not necessarily require fasting^23^, and the Friedewald equation is valid in patients with TG < 400 even without fasting^23^. Indeed, we found no difference in the prognosis of patients with high LLRA with or without fasting before blood sampling (Supplemental Fig. 11).

In summary, high HDL is an independent factor for poor prognosis in ALS. The predictive role of low LDL is applicable only in women. Since HDL and LDL decrease as the disease progresses, an earlier assessment of lipid profiles is required to evaluate the prognosis. Moreover, our study highlights that HDL is a possible pathogenic factor, and further research is necessary to confirm whether it could be a therapeutic target.

## Supplementary Information

Supplementary Information.

## Data Availability

The datasets generated and/or analyzed during the current study are available from the corresponding author upon reasonable request.
