# Supplementary material for: Investigation of the prognostic predictive value of serum lipid profiles in amyotrophic lateral sclerosis: roles of sex and hypermetabolism
Source: Sci Rep. 2022 Feb 3;12:1826. doi: 10.1038/s41598-022-05714-w (PMC8814149; doi:10.1038/s41598-022-05714-w)
Supplement: Supplementary file 1 — Supplementary Information. [file 41598_2022_5714_MOESM1_ESM.pdf]

Title: Investigation of the prognostic predictive value of serum lipid profiles in amyotrophic lateral sclerosis: roles of sex and hypermetabolism

Journal of neurology

Ryutaro Nakamura, MD[1]; Mika Kurihara, MS[2]; Nobuhiro Ogawa, MD, PhD[1]; Akihiro Kitamura, MD, PhD[1]; Isamu Yamakawa, MD, PhD[1]; Shigeki Bamba, MD, PhD[2]; Mitsuru Sanada, MD, PhD[1]; Masaya Sasaki, MD, PhD[2]; Makoto Urushitani, MD, PhD[1].

[1]Department of Neurology and [2] Division of Clinical Nutrition, Shiga University of Medical Science, Tsukinowa, Seta, Shiga, Japan

Correspondence to: Makoto Urushitani, MD, Ph.D.
Department of Neurology, Shiga University of Medical Science, Tsukinowa, Seta, Shiga, Japan
Telephone/Fax: +81 (0) 77 548 2160
E-mail: uru@belle.shiga-med.ac.jp

Supplemental Fig.1

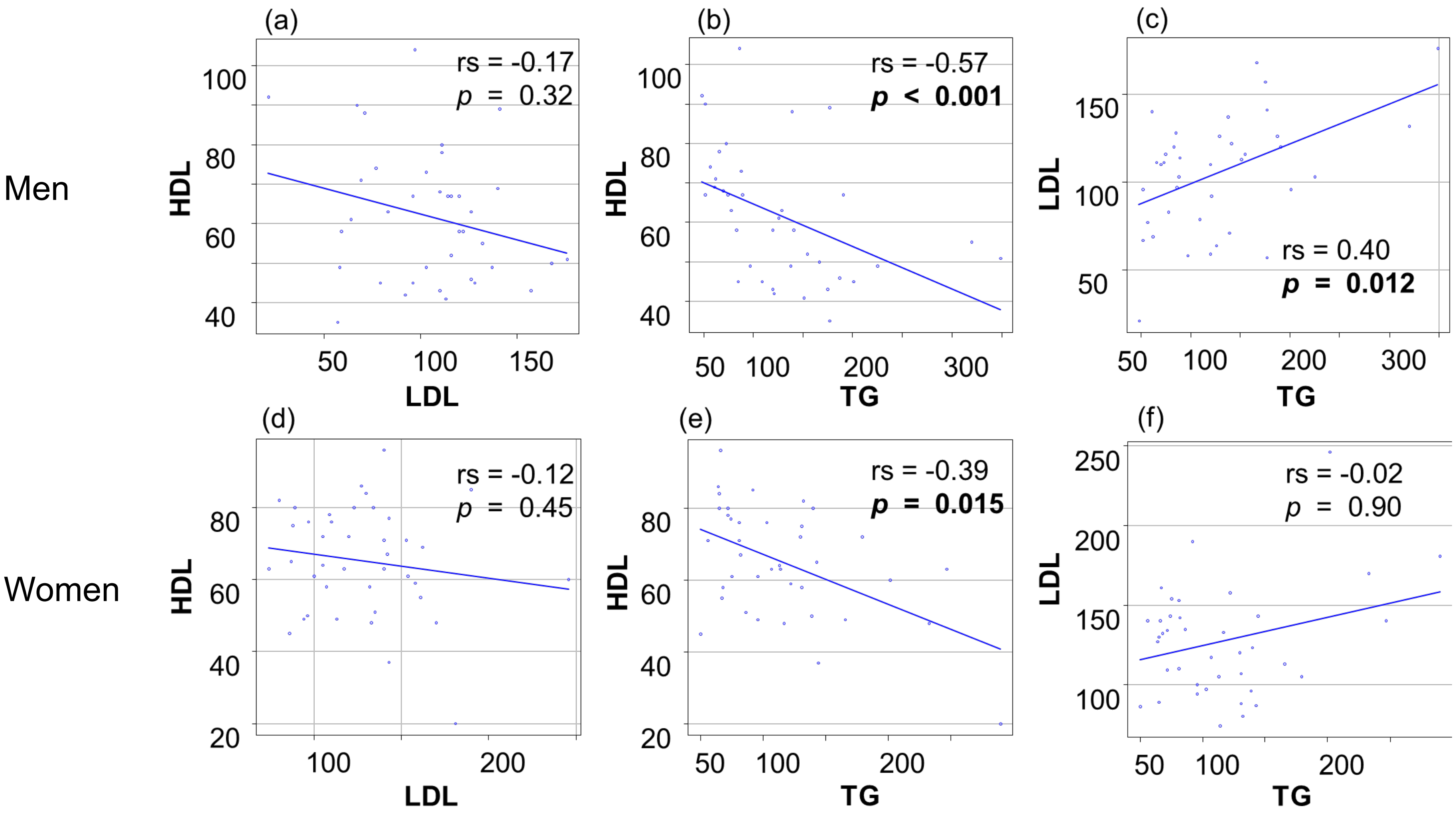

Supplemental Fig.1 The relationship among lipid profiles in each sex. HDL was negatively associated with TG in both sex (b, e), and LDL did positively with TG in males(c). There was no relationship between HDL and LDL in both sex (a,d), and between LDL and TG in females (f). Rs means Spearman's rank-correlation coefficient. P-value is based on Spearman's test.

Supplemental Fig.2

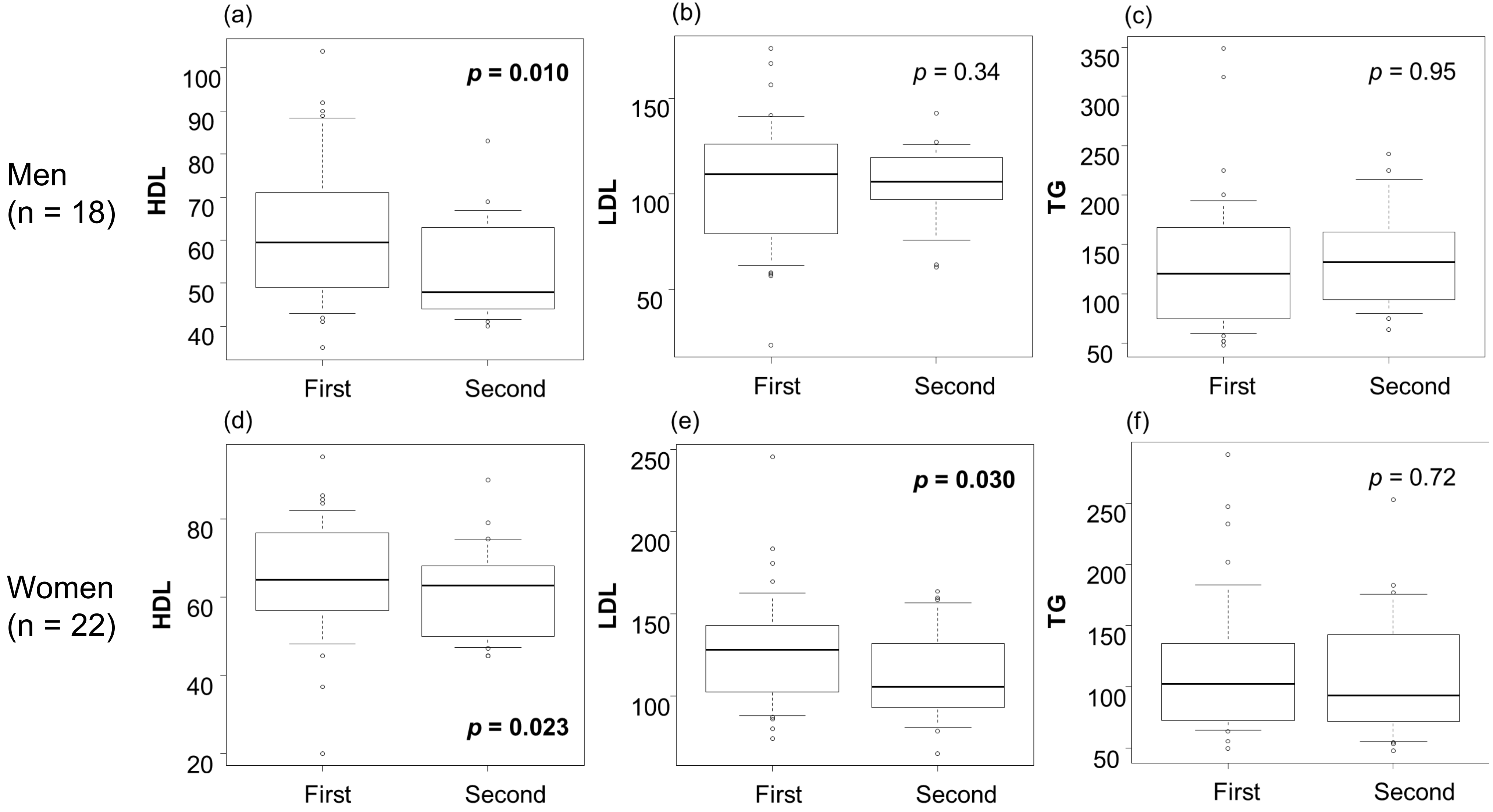

Supplemental Fig.2 Comparison of lipid profiles between the first blood test and the follow-up blood test. P-value is based on Wilcoxon signed-rank test. HDL significantly decreased in both sex (a,d), and LDL did in females (e). The others did not significantly change (b,c,f).

Supplemental Fig.3

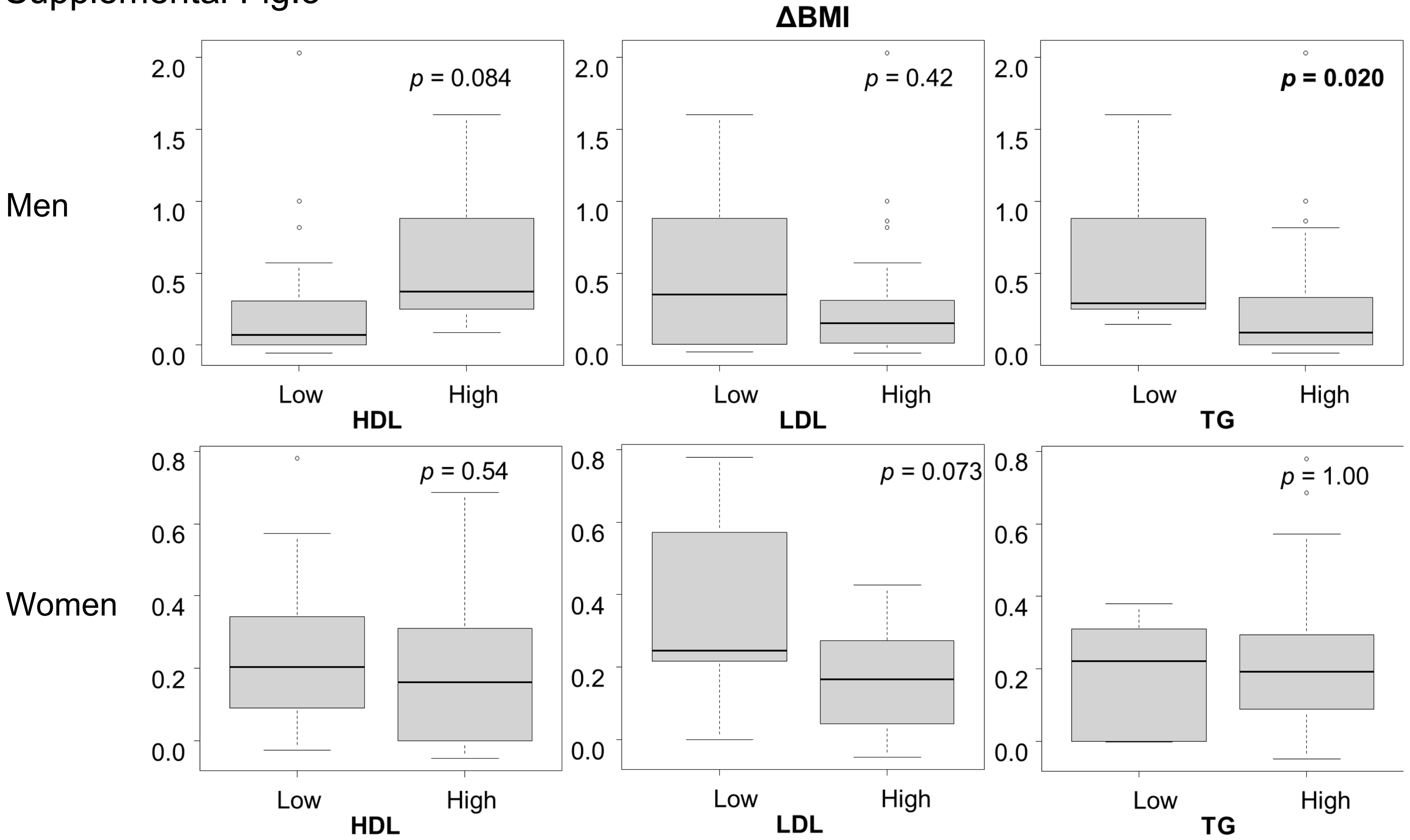

Supplemental Fig.3 The relationship between lipid profiles and ΔBMI/month in each sex. Mann–Whitney U test showed that the decline rate of BMI was significantly higher in males with low TG (c). Males with high HDL (a) and females with low LDL (e) demonstrated the same trend, although not significant. There was no significant relationship in the other groups (b, d, f).

Supplemental Fig.4

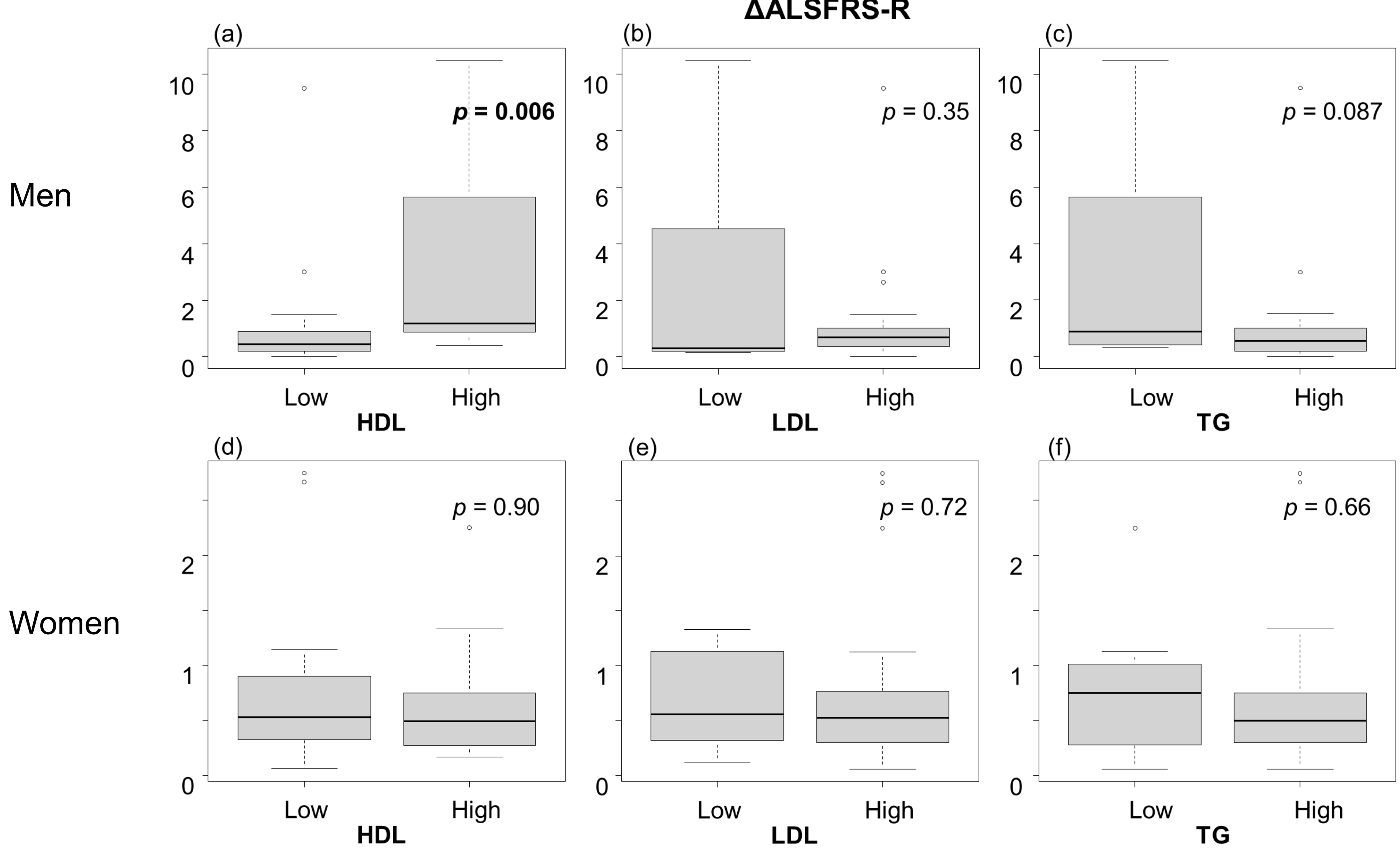

Supplemental Fig.4 The relationship between lipid profiles and ΔALSFRS-R in each sex. Mann–Whitney U test showed that ΔALSFRS-R was significantly higher in males with high HDL (a). Males with low TG showed the same trend, although not significant (c). There was no significant relationship in the other groups (b, d, e, f).

Supplemental Fig.5

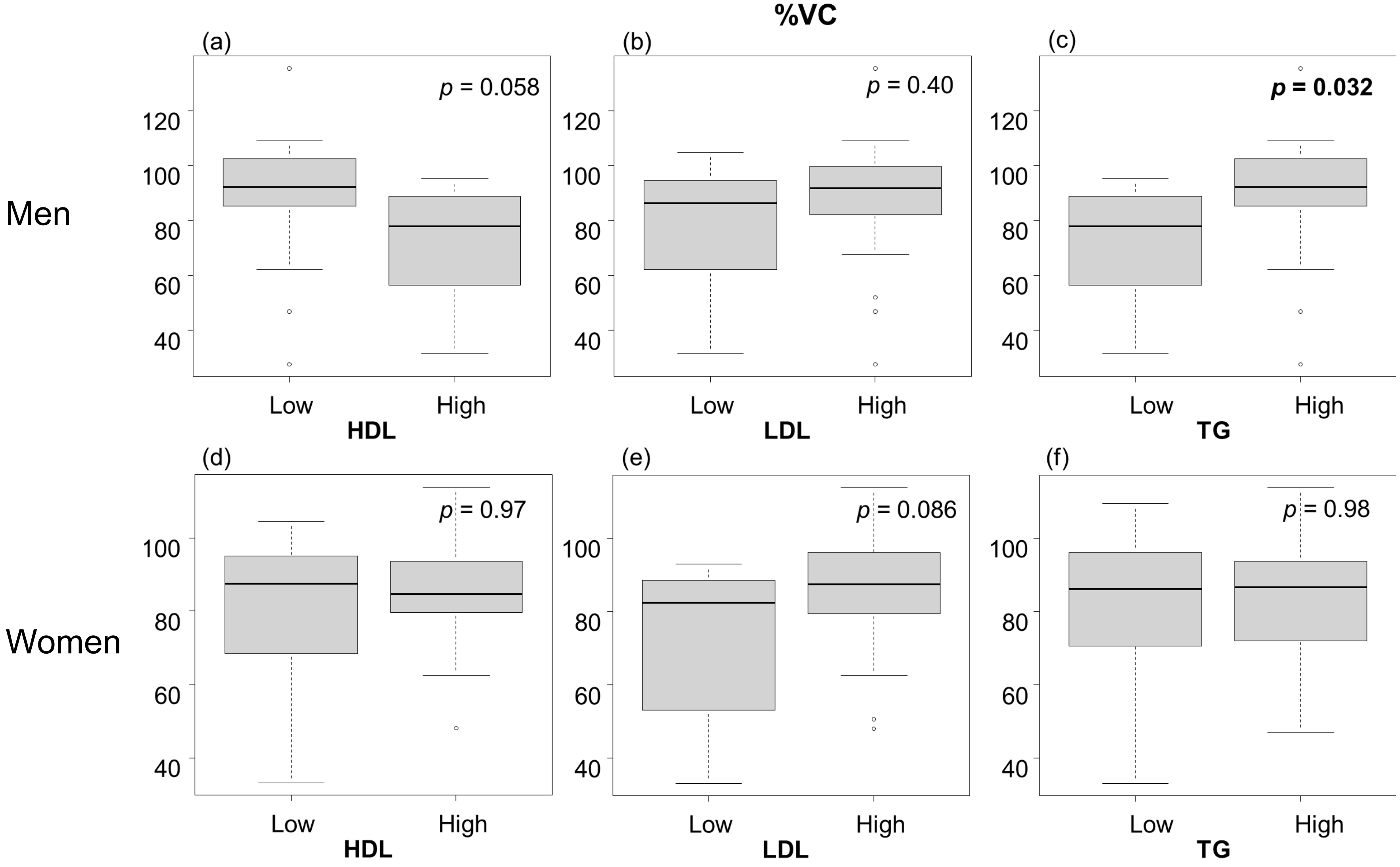

Supplemental Fig.5 The relationship between lipid profiles and %VC in each sex. Mann–Whitney U test showed that %VC was significantly lower in males with low TG (c). This trend was also seen in males with high HDL (a) and females with low LDL (d), although not significant. There was no significant relationship in the other groups (b, e, f).

Supplemental Fig.6

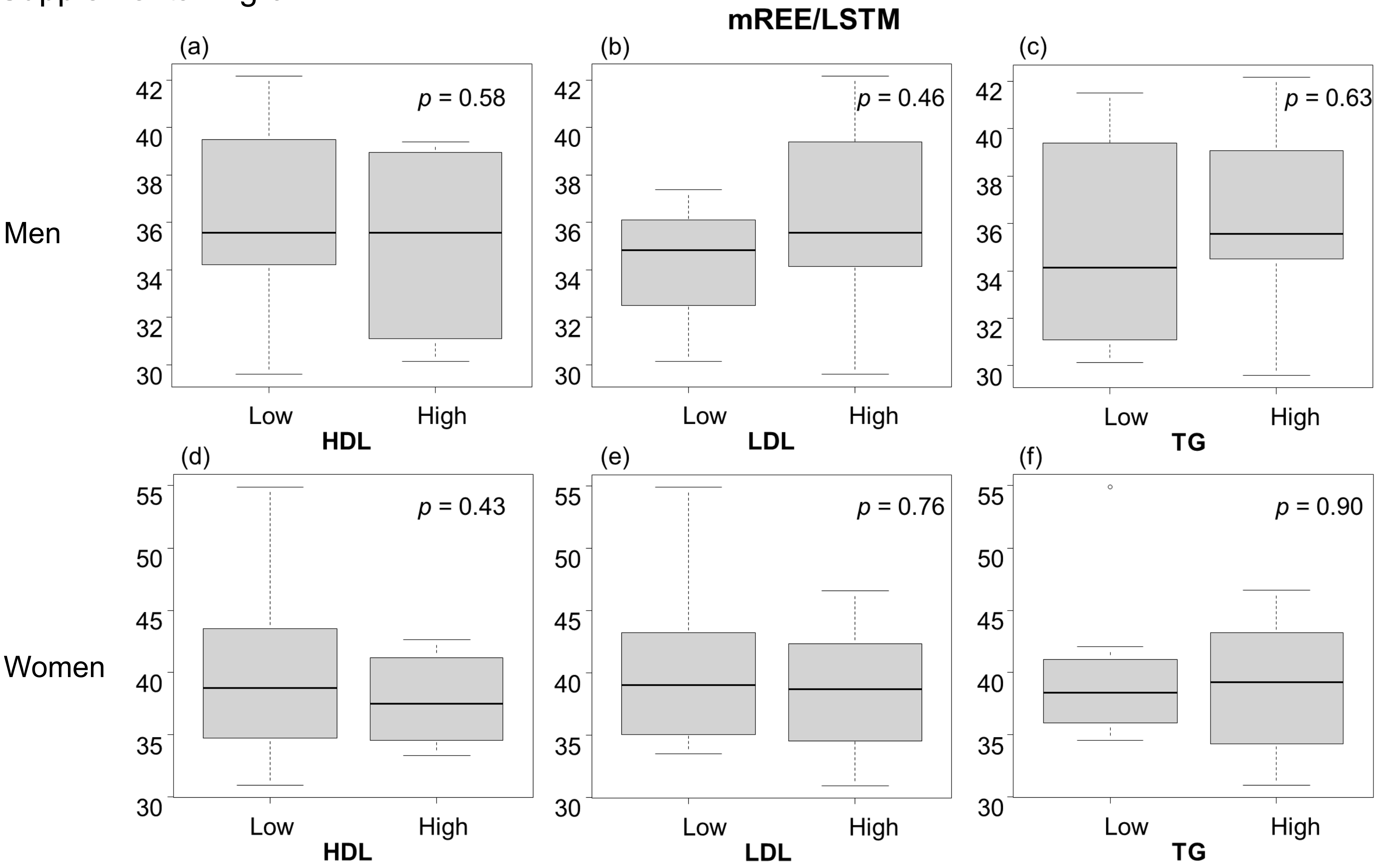

Supplemental Fig.6 The relationship between lipid profiles and mREE/LSTM in each sex. There was no significant relationship between them in all groups (a- f).

Supplemental Fig.7

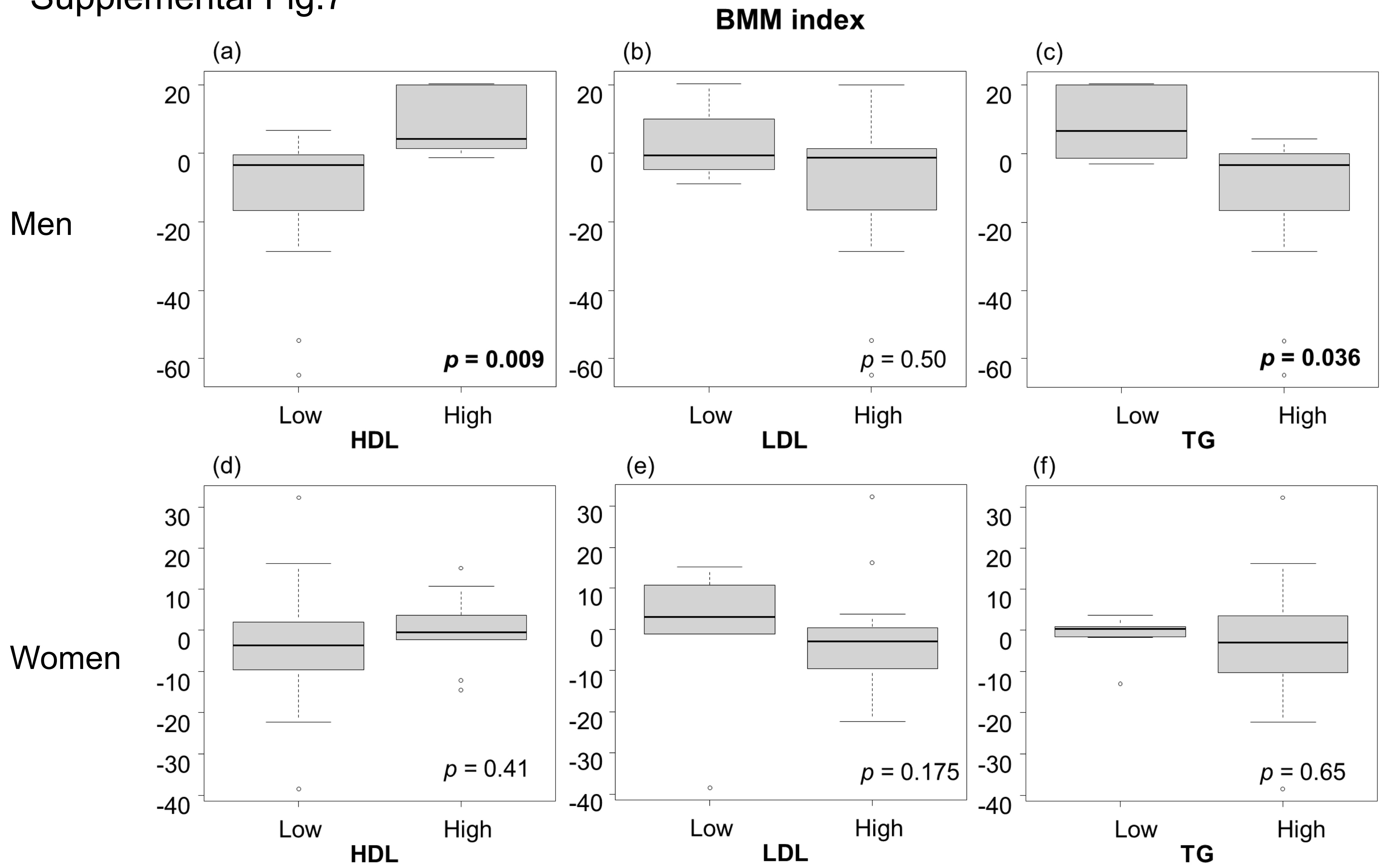

Supplemental Fig.7 The association between serum lipids and BMI-muscle metabolism index (BMM index) in each sex. BMM index was significantly higher in males with high HDL (a) and low TG (c). There was no significant relationship in the other groups (b, d, e, f).

Supplemental Fig.8

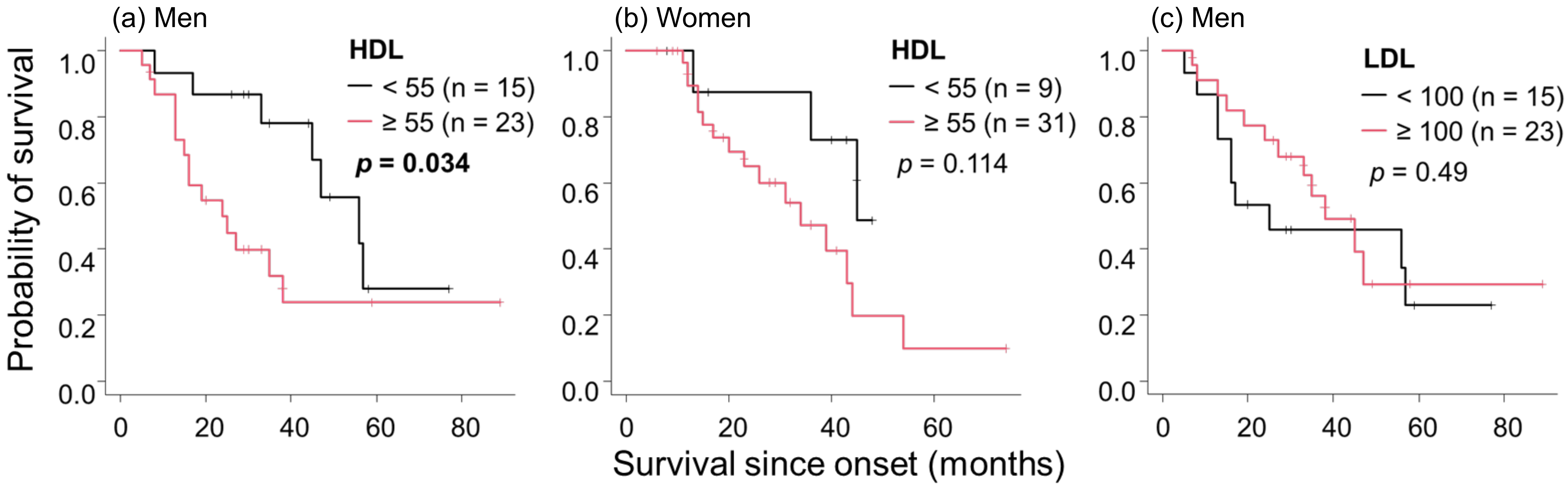

Supplemental Fig.8 Comparison of lipid profiles and the survival at the cut-off value similar to the study of Ingre, et al. Males with HDL ≥ 55 mg/dl had longer survival than those with HDL < 44 mg/dl (a). Females showed the same trend, although not reaching significance (b). Males with LDL < 100 mg/dl did not have a significant shorter survival than those with LDL ≥ 100mg/dl (c).

Supplement Fig.9

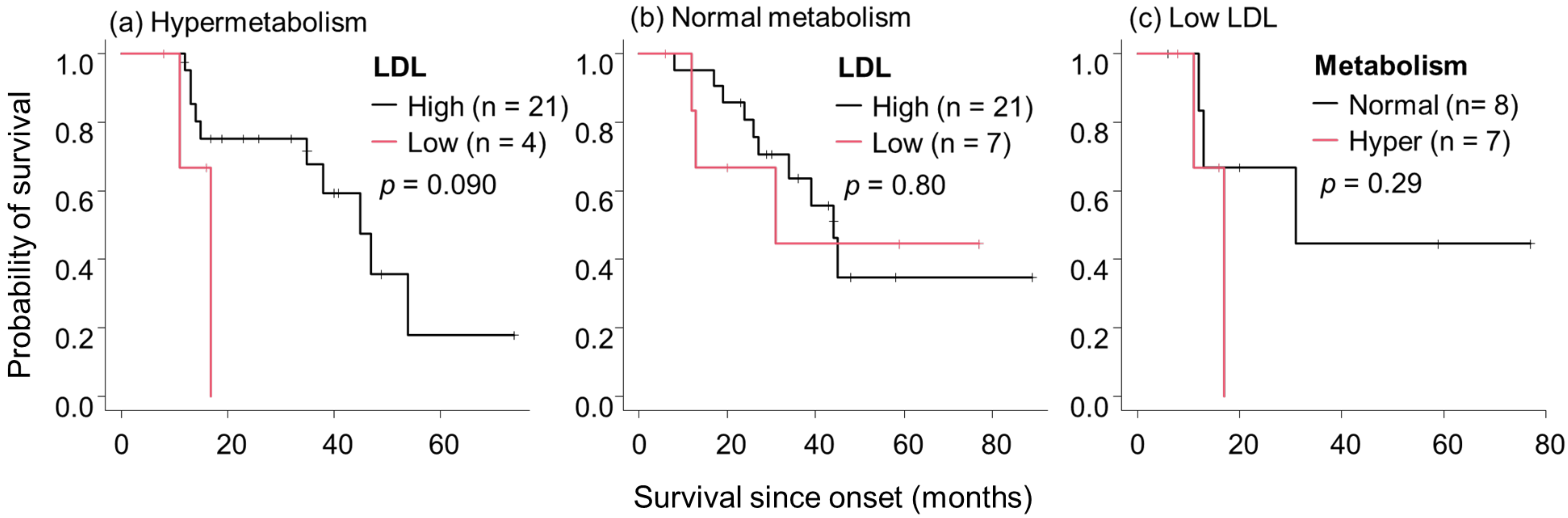

Supplement Fig.9 The relationship between LDL and hypermetabolism on the survival. Kaplan-Meier analyses and the log-rank tests showed that patients with low LDL had a shorter survival than patients with high LDL in patients with hypermetabolism (a), although not significant, and did not in the patients with normal metabolism (b). Patients with hypermetabolism did not have a significantly shorter survival than patients with normal metabolism in the low LDL group (c).

Supplement Fig.10

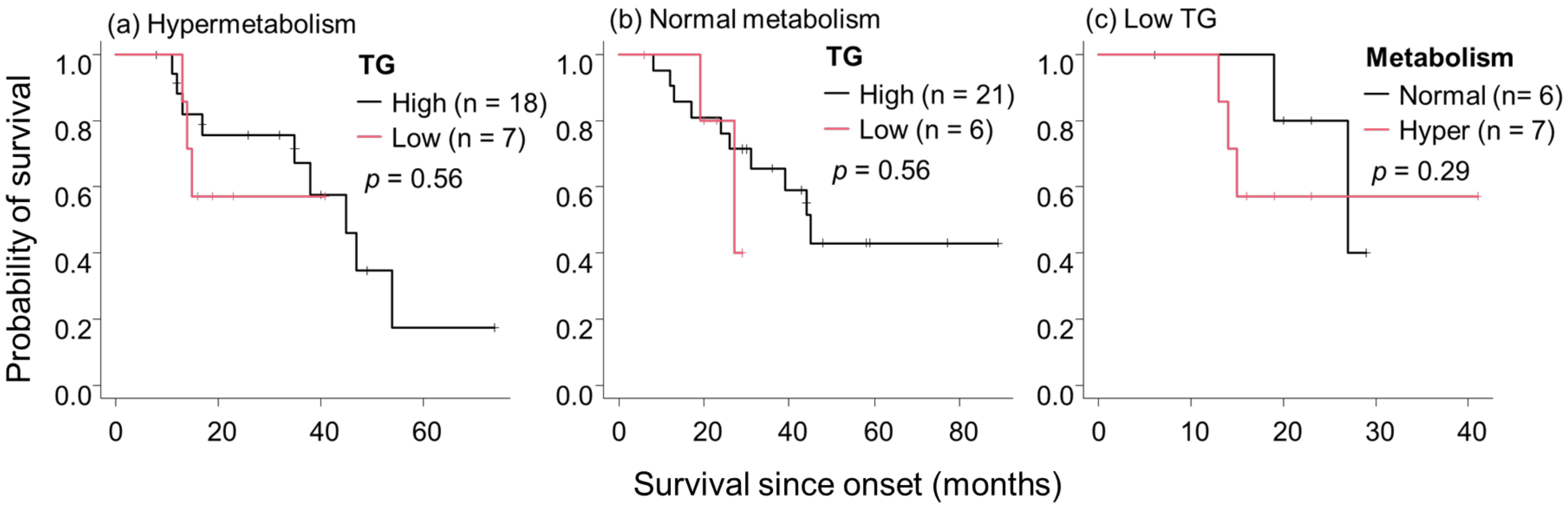

Supplement Fig.10 The relationship between TG and hypermetabolism on the survival. Kaplan-Meier analyses and the log-rank tests showed that patients with low TG did not have a shorter survival in patients with hypermetabolism (a) and normal metabolism (b). In the low TG group, patients with hypermetabolism did not have a significantly shorter survival than patients with normal metabolism (c).

Supplemental Fig.11

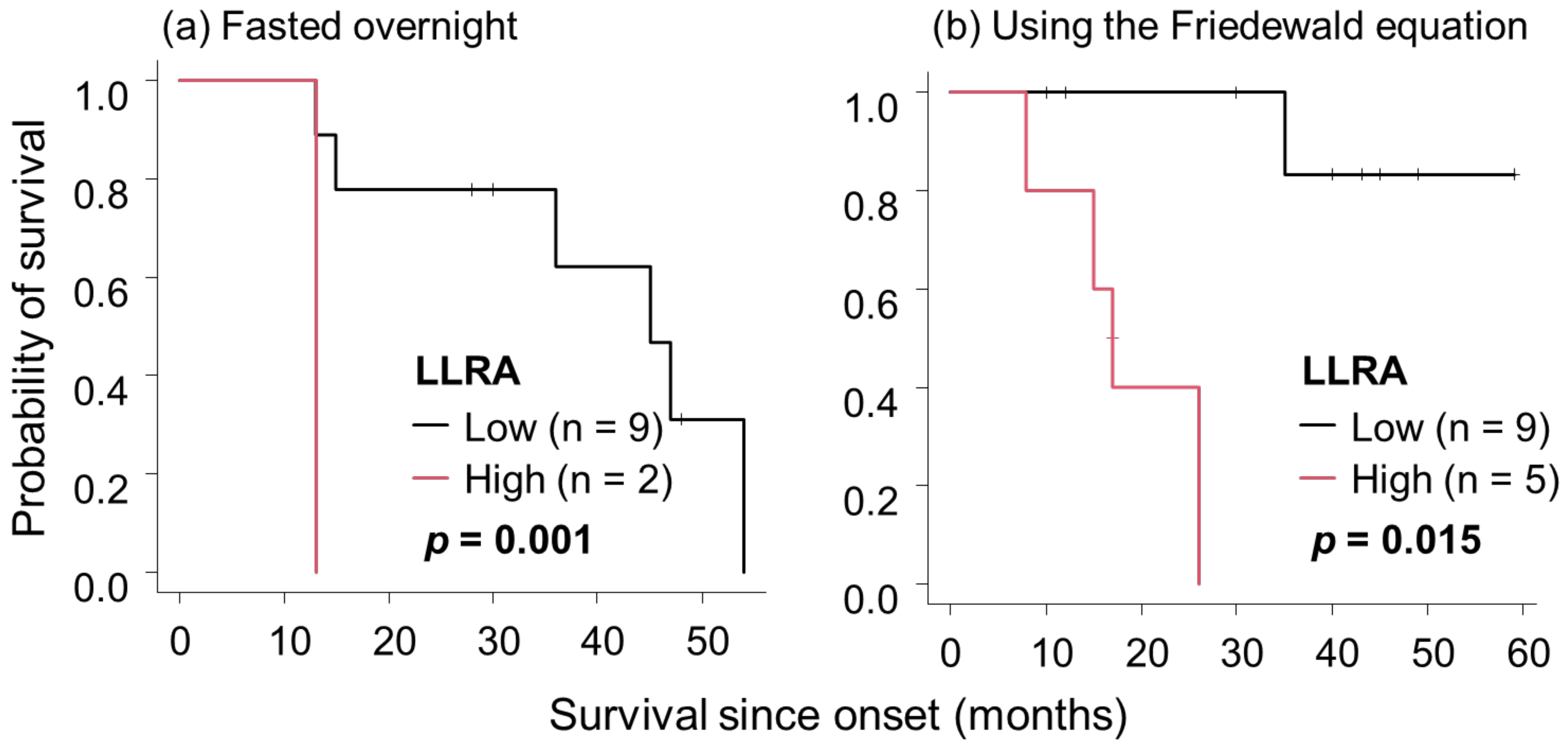

Supplemental Fig.11 Significance of lipid-linked risk for ALS (LLRA) as a predictive factor for poor prognosis. in patients fasted overnight or using Friedewald equation. Kaplan-Meier analyses and the log-rank tests showed that high lipid-linked risk for ALS (LLRA) was also a significant poor prognostic factor in patients fasted overnight (a) and patients using Friedewald equation (b), although the number was very small.

Supplement Fig.12

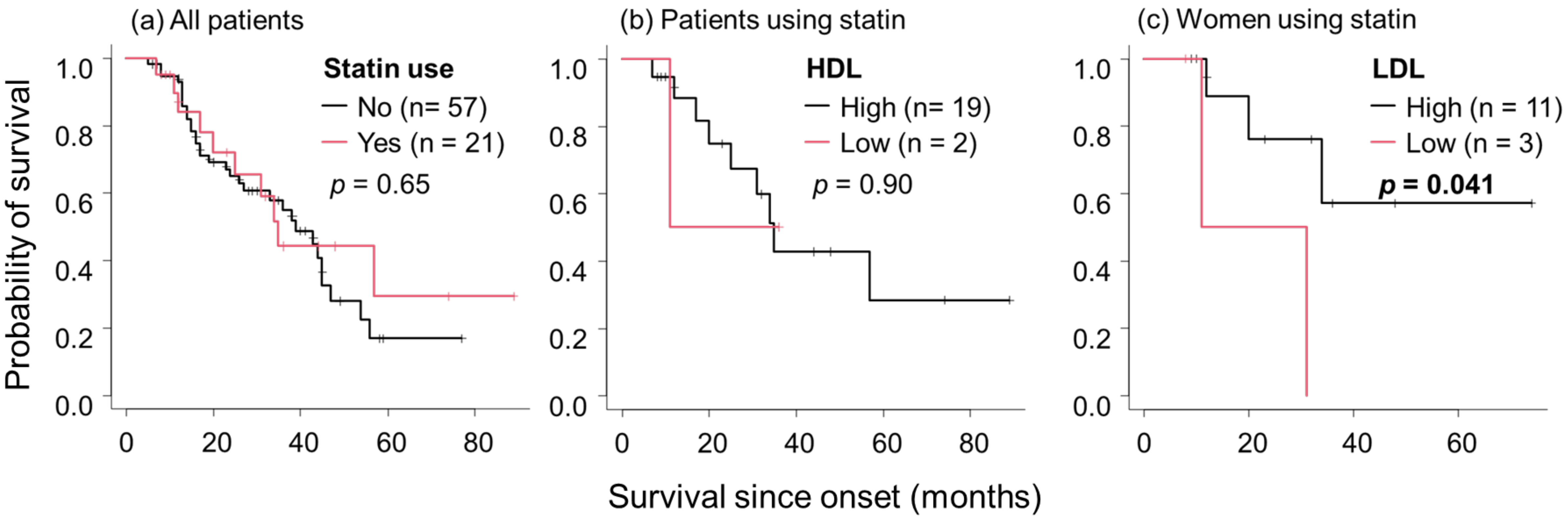

Supplemental Fig.12 The effect of statin on the survival. Kaplan-Meier analyses and the log-rank test showed that the survival did not differ regardless of using statin or not (a). Patients with high HDL did not have a significantly shorter survival in patients using statin (b). Low LDL was also a significant poor prognostic factor in women using statin as well as without statin (c).

Supplemental Table. Lipid profiles in patients stratified by using statin

	Men			Women		
	using statin	without statin	*p**	using statin	without statin	*p**
HDL (mg/dl)	58 [47, 60]	63 [49, 74]	0.169	64 [58, 72]	66 [53, 80]	0.54
LDL (mg/dl)	114 [78, 118]	110 [81, 127]	0.57	109 [101, 133]	135 [110, 143]	0.169
TG (mg/dl)	126 [105, 148]	109 [72, 171]	0.46	114 [81, 131]	94 [72, 143]	0.62

	All		
	using statin	without statin	*p**
High HDL	2/21 (10%)	21/57 (37%)	**0.024**
Low LDL	5/21 (24%)	14/57 (25%)	1
Low TG	2/20 (10%)	18/57 (32%)	0.077
High LLRA	4/21 (19%)	27/57 (47%)	**0.036**

Data represent the median value [interquartile range]. *$p < 0.05$, by Fisher's exact test or the Mann–Whitney U test.

HDL, high-density lipoprotein; LDL, low-density lipoprotein; TG, triglycerides.
